# Supplementary material for: Morphological Characteristics and Comparative Chloroplast Genome Analyses between Red and White Flower Phenotypes of Pyracantha fortuneana (Maxim.) Li (Rosaceae), with Implications for Taxonomy and Phylogeny
Source: Genes (Basel). 2022 Dec 18;13(12):2404. doi: 10.3390/genes13122404 (PMC9778009; doi:10.3390/genes13122404)
Supplement: Supplementary file 1 [file genes-13-02404-s001.zip › genes-2084829-supplementary.pdf]

Supplementary Materials

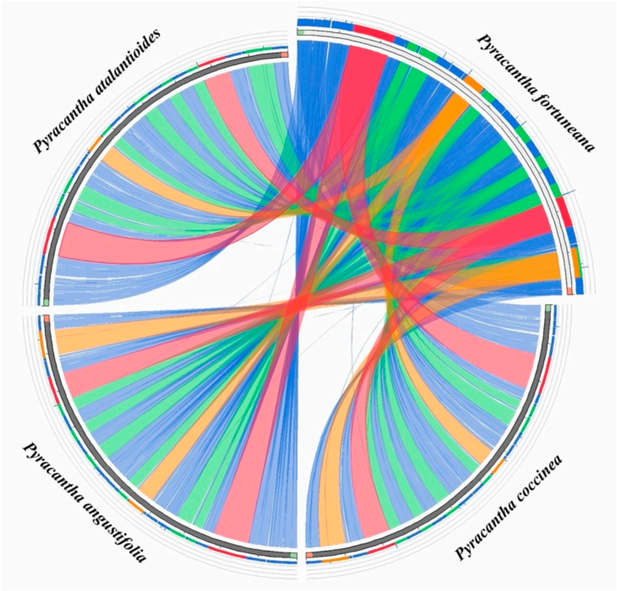

Figure S1. Synteny comparison among the chloroplast genomes of *P. fortuneana* and another three *Pyracantha* species. The colored blocks outside the sequences refer to the score/max bit core ration, with red > 0.75, orange > 0.50, green > 0.25, and blue ≤ 0.25.

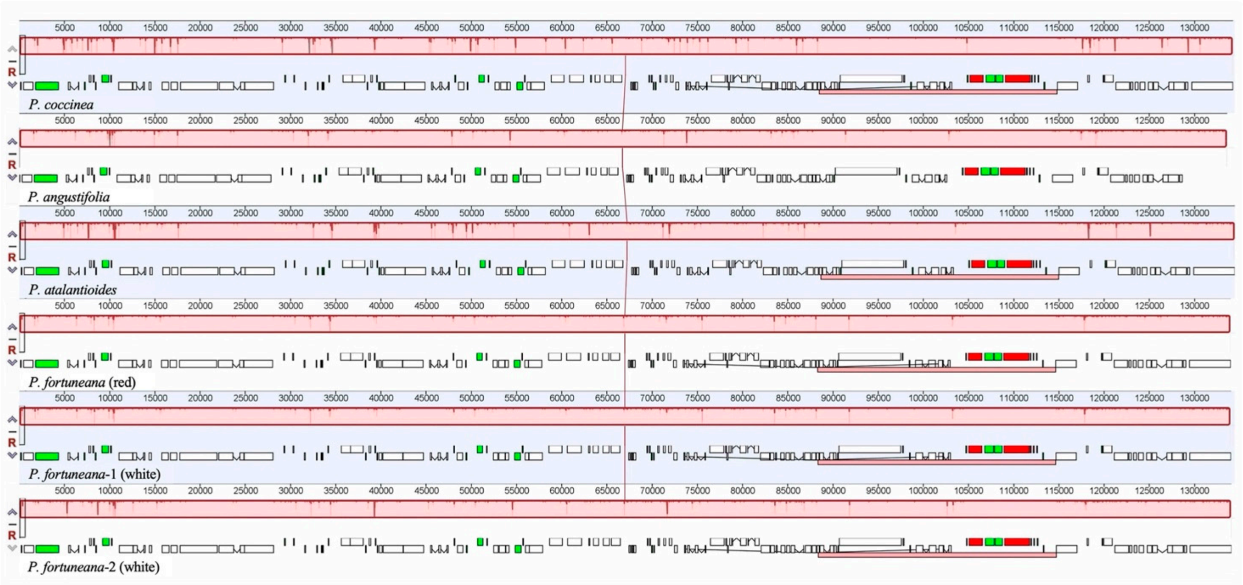

Figure S2. The structural rearrangement analysis of six *Pyracantha* chloroplast genomes using the mauve multiple alignment algorithm.

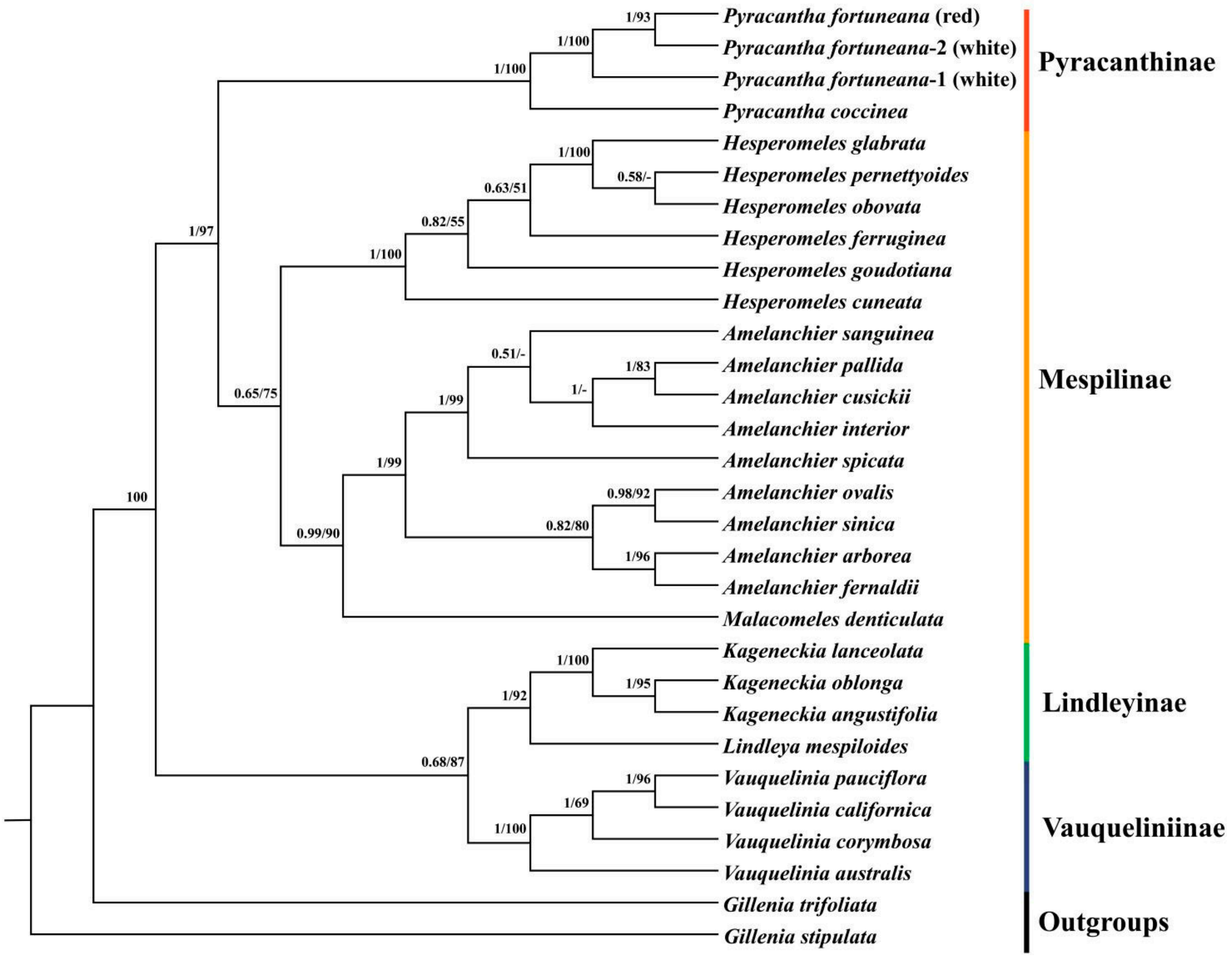

Figure S3. The phylogenetic tree of the genus *Pyracantha* based on 30 nrDNA ITS sequences using both ML and BI methods. The BI posterior probabilities / ML bootstrap values are displayed above the lines ("-" stands for the value less than 0.5/50).

Table S1. Sample list of chloroplast genomes used in this study.

Species Name	Accession	Species Name	Accession
Amelanchier sinica	MK920291	*Pyracantha fortuneana*-1 (white)	NC_059101
Malacomeles denticulata	NC_045325	*Pyracantha fortuneana*-2 (white)	MW596361
Hesperomeles ferruginea	NC_045328	*Pyracantha atalantioides*	MW801001
Hesperomeles glabrata	MK920298	*Vauquelinia australis*	NC_045309
Hesperomeles obovata	MK920299	*Vauquelinia californica*	NC_062344
Hesperomeles goudotiana	NC_045327	*Vauquelinia californica*-1	KY419925
Hesperomeles pernettyoides	NC_045329	*Vauquelinia californica*-2	MN068269
Crataegus cuneata	NC_058896	*Vauquelinia corymbosa*	MN068249
Crataegus pinnatifida	NC_057086	*Kageneckia angustifolia*	NC_045322
Crataegus hupehensis	NC_054155	*Kageneckia crataegifolia*	KY420027
Crataegus laevigata	NC_062347	*Kageneckia oblonga*	NC_045324
Crataegus rhipidophylla	NC_062345	*Kageneckia lanceolata*	MN068265
Crataegus kansuensis	NC_039374	*Lindleya mespiloides*	MN068248
Pyracantha coccinea	NC_062343	*Gillenia stipulata*	NC_045321
Pyracantha angustifolia	KY419957	*Gillenia trifoliata*	NC_045311

Table S2. Sample list of rDNA ITS sequences used in this study.

Species Name	Accession	Species Name	Accession
Amelanchier sanguinea	MN905587	*Lindleya mespiloides*	MN577906
Amelanchier pallida	MN905586	*Kageneckia angustifolia*	MN905589
Amelanchier interior	MN905585	*Kageneckia oblonga*	MN577932
Amelanchier fernaldii	MN905583	*Kageneckia lanceolata*	MN068265
Amelanchier cusickii	MN905582	*Malacomeles denticulata*	MN905591
Amelanchier arborea	MN905579	*Pyracantha fortuneana* (red)	OP821228
Amelanchier ovalis	MN215988	*Pyracantha fortuneana*-1 (white)	MN216013
Amelanchier sinica	MN216016	*Pyracantha fortuneana*-2 (white)	SRR17631715
Amelanchier spicata	MN215976	*Pyracantha coccinea*	SRR13004386
Hesperomeles pernettyoides	MN905597	*Vauquelinia californica subsp. sonorensis*	MN905593
Hesperomeles ferruginea	MN905596	*Vauquelinia pauciflora*	MN905577
Hesperomeles goudotiana	MN905595	*Vauquelinia corymbosa* subsp. *heterodon*	MN905576
Hesperomeles cuneata	MN905594	*Vauquelinia australis*	MN577917
Hesperomeles obovata	MN216004	*Gillenia stipulata*	MN905588
Hesperomeles glabrata	MN216003	*Gillenia trifoliata*	MN577923

Table S3. The codon number in six *Pyracantha* chloroplast genomes.

Species	Codons
Pyracantha atalantioides	26,288
Pyracantha angustifolia	26,317
Pyracantha coccinea	26,291
Pyracantha fortuneana (red)	26,292
Pyracantha fortuneana-1 (white)	26,291
Pyracantha fortuneana-2 (white)	26,292

Table S4. List of the potential RNA editing sites in the chloroplast protein-coding genes of *Pyracantha fortuneana* (red) and two individuals of *P. fortuneana* (white).

Nt Pos	AA Pos	Align Col	Effect	Score	Gene
64	22	22	CGG (R) => TGG (W)	1	*accD*
1463	488	519	CCT (P) => CTT (L)	1	*accD*
773	258	258	TCA (S) => TTA (L)	1	*atpA*
914	305	305	TCA (S) => TTA (L)	1	*atpA*
1148	383	383	TCA (S) => TTA (L)	1	*atpA*
37	13	13	CCT (P) => TCT (S)	1	*atpB*
629	210	213	TCA (S) => TTA (L)	1	*atpI*
559	187	187	CAT (H) => TAT (Y)	1	*clpP*
634	212	227	CAT (H) => TAT (Y)	1	*matK*
149	50	50	TCA (S) => TTA (L)	1	*ndhB*
467	156	156	CCA (P) => CTA (L)	1	*ndhB*
542	181	181	ACG (T) => ATG (M)	1	*ndhB*
586	196	196	CAC (H) => TAC (Y)	1	*ndhB*
737	246	246	CCA (P) => CTA (L)	1	*ndhB*
746	249	249	TCT (S) => TTT (F)	1	*ndhB*
830	277	277	TCA (S) => TTA (L)	1	*ndhB*
1112	371	371	TCA (S) => TTA (L)	1	*ndhB*
1255	419	419	CAT (H) => TAT (Y)	1	*ndhB*
1481	494	494	CCA (P) => CTA (L)	1	*ndhB*
383	128	128	CCA (P) => CTA (L)	1	*ndhD*
599	200	200	TCA (S) => TTA (L)	1	*ndhD*
674	225	225	TCA (S) => TTA (L)	1	*ndhD*
878	293	293	TCA (S) => TTA (L)	1	*ndhD*
290	97	97	TCG (S) => TTG (L)	1	*ndhF*
392	131	131	TCC (S) => TTC (F)	1	*ndhF*
1876	626	631	CTT (L) => TTT (F)	1	*ndhF*
418	140	140	CGG (R) => TGG (W)	1	*petB*
611	204	204	CCA (P) => CTA (L)	1	*petB*
214	72	72	CCT (P) => TCT (S)	1	*psbE*
77	26	26	TCT (S) => TTT (F)	1	*psbF*
2	1	1	ACG (T) => ATG (M)	1	*psbL*
830	277	279	TCA (S) => TTA (L)	1	*rpoA*
338	113	113	TCT (S) => TTT (F)	1	*rpoB*
566	189	190	TCG (S) => TTG (L)	1	*rpoB*
1994	665	684	TCT (S) => TTT (F)	1	*rpoB*
248	83	83	TCA (S) => TTA (L)	1	*rps2*
80	27	27	TCA (S) => TTA (L)	1	*rps14*
149	50	53	CCA (P) => CTA (L)	1	*rps14*

Table S5. Summary of SSRs in six *Pyracantha* chloroplast genomes.

Repeat types	A/T	C/G	AG/ CT	AT/ AT	AAAT /ATTT	AATT/ AATT	AAGG C/CCTT G	AATCC/ ATTGG	AATATT/ AATATT	AACTAT/ AGTTAT	Total
3					7			1			8
5			1	15							16
6				1							1
7				1							1
10	23										23
11	11	2									13
12	13										13
13	7										7
14	3	1									4
15	4										4
16	1										1
17	2										2
18	2										2
19	1										1
25	1										1
Total	68	3	1	17	7	0	0	1	0	0	97
Pyracantha angustifolia											
3					7	1		1		1	10
5			1	22							23
6				4							4
10	21	2									23
11	9	1									10
12	13										13
13	5										5
14	5	1									6
15	2										2
16	5	1									6
17	5										5
18	1										1
20	1										1
24	1										1
34	1										1
Total	69	5	1	26	7	1	0	1	0	1	111
Pyracantha atalantioides											
3					6	1		1	1		9
4							2				2
5			1	20							21
6				4							4
10	21										21
11	11										11

12	10	2									12
13	3										3
14	7										7
15	8										8
16	1										1
17	3										3
18	1										1
19	2										2
Total	67	2	1	24	6	1	2	1	1	0	105
Pyracantha coccinea											
3					7			1			8
5			1	20							21
6				3							3
7				1							1
10	20	3									23
11	8										8
12	12										12
13	6										6
14	4										4
15	2										2
16	5										5
17	3										3
18	1										1
19	2										2
22	1										1
23	1										1
Total	65	3	1	24	7	0	0	1	0	0	101
Pyracantha fortuneana (red)											
3					7			1			8
5			1	20							21
6				3							3
7				1							1
10	20	2									22
11	8	1									9
12	13										13
13	5										5
14	3										3
15	2										2
16	6										6
17	4										4
18	1										1
19	1										1
21	1										1
23	1										1
Total	65	3	1	24	7	0	0	1			101

Pyracantha fortuneana-1 (white)

3					7			1			8
5			1	23							24
6				2							2
7				1							1
10	19	2									21
11	11	1									12
12	16										16
13	2										2
14	5	1									6
15	4										4
16	5										5
17	1										1
20	1										1
21	2										2
22	1										1
Total	67	4	1	26	7	0	0	1	0	0	106

Pyracantha fortuneana-2 (white)

Table S6. The neutral selected sites detected in the 78 CDSs of six *Pyracantha* chloroplast genomes.

Rate estimate	LRT	p-value	Gene	Region	Rate estimate	LRT	p-value	Gene	Region
189.581	1.307	0.2529	*accD*	LSC	279.163	0.513	0.4739	*petB*	LSC
337.637	1.081	0.2984			277.363	0.631	0.4268		
110.059	1.415	0.2342	*atpA*	LSC	281.809	-0.045	1	*petL*	LSC
118.066	1.91	0.167			148.181	0.403	0.5255		
118.104	1.756	0.1852	*ccsA*	SSC	219.006	0.483	0.4872	*psaB*	LSC
196.862	2.192	0.1387	*cemA*	LSC	240.572	0.369	0.5434		
202.472	0.223	0.6369	*matK*	LSC	285.467	1.792	0.1807	*psbA*	LSC
138.283	0.377	0.539			254.254	2.681	0.1015	*psbC*	LSC
249.616	-0.001	1			322.933	0.631	0.4271	*psbK*	LSC
140.031	1.352	0.245	*ndhA*	SSC	221.789	2.031	0.1542	*rpl14*	LSC
229.471	0.5	0.4794	*ndhC*	SSC	241.761	0.824	0.3639	*rpl32*	SSC
174.035	0.62	0.4309	*ndhD*	SSC	196.116	1.123	0.2892	*rpl33*	LSC
161.961	1.09	0.2965			368.073	0.736	0.3911	*rpoA*	LSC
141.525	0.427	0.5136			194.096	2.568	0.1091	*rps11*	LSC
157.918	0.951	0.3294			199.376	1.308	0.2528	*rps12*	IR
0.008	-0.034	1	*ndhF*	SSC	342.758	0.681	0.4094	*rps15*	SSC
219.006	0.483	0.4872			266.225	0.556	0.456	*rps18*	LSC
324.996	0.579	0.4467			309.785	2.234	0.135	*ycf2*	IR
374.718	2.502	0.1137			271.046	0.535	0.4646	*ycf3*	LSC
250.479	1.343	0.2465			264.924	0.514	0.4733	*rpoC2*	LSC
238.061	0.599	0.4389	*ndhJ*	LSC	468.867	0.432	0.511		
